# Intestinal Helminth Infections and Associated Risk Factors among School-Aged Children of Bamendjou Community, West Region of Cameroon

**DOI:** 10.1155/2021/6665586

**Published:** 2021-04-23

**Authors:** Matsinkou Mba Rosine Ruth, Yamssi Cedric, Mbong Erica Malla, Noumedem Anangmo Christelle Nadia, Tateng Ngouateu Aime, Megwi Leonelle, Vincent Khan Payne

**Affiliations:** ^1^Department of Animal Biology, Faculty of Science, University of Dschang, P.O. Box 067, Dschang, Cameroon; ^2^Department of Biomedical Sciences, Faculty of Health Sciences, University of Bamenda, P.O. Box 39, Bambili, Cameroon; ^3^Department of Microbiology, Hematology and Immunology Faculty of Medicine and Pharmaceutical Sciences, University of Dschang, P.O. Box 96, Dschang, Cameroon

## Abstract

**Background:**

Infection with intestinal nematodes is of major public health concern worldwide, and school-age children and pregnant women are the principal victims. The present study was undertaken to provide baseline information on the status of gastrointestinal nematodes among school-age children in Bamendjou. *Material and Methods*. Four hundred and ninety-three (493) stool samples were collected from school children in six (6) different schools (two nursery, two primary, and two secondary schools). Qualitative and quantitative analyses of stool samples were done using the simple flotation and McMaster count techniques, respectively.

**Results:**

Among the 493 participants, 57 (11.6%) stool samples were positive for at least one nematode species. Four nematodes are as follows: *Ascaris* sp., *Trichuris* sp., hookworms, and *Strongyloides* sp. with respective prevalence and intensities of infection of 6.1% and 2260 ± 6377.98, 3.4% and 223.53 ± 264.054, 3.0% and 416.67 ± 427.061, and 0.2% and 200 ± 00, respectively. The data on the prevalence of nematodes with respect to sex showed that females (13.1%) were more infected than males (12.2%) (*P* > 0.05). Furthermore, with respect to age, older children were more infected than younger ones. Cases of double parasitism were encountered with a prevalence of 1.2%. According to the fecal concentration of eggs, 61.90% of the infections were light. Risk factors such as drinking water from streams and not wearing shoes all the time were significant with infections.

**Conclusion:**

The relatively low overall prevalence (11.6%) obtained in this study shows that the national deworming campaign is proving effective, though a more holistic approach is required to prevent infections from bouncing back after such campaigns.

## 1. Introduction

Intestinal parasitic diseases constitute a major problem in public health in tropical and subtropical zones where climatic factors such as humidity, temperature, lack of hygiene, poverty, demographic conditions, and socio-political instability favor their development [1]. Parasitic groups leading to these intestinal parasitic infections are the protozoans and helminths. It is estimated that soil-transmitted helminths (STHs) affect more than 2 billion people worldwide, and the greatest number of infections occurs in sub-Saharan Africa, South and Central America, and China and East Asia [2]. In Cameroon, soil-transmitted helminths are widely distributed all over the country, and it is estimated that more than 10 million people are infected [3]. According to Brooker et al. [4], 5.6 million people were infected with *A. lumbricoides*, 6.5 million with *T. trichiura*, and 2.6 million with hookworms in Cameroon. These infections are among the most common and neglected public health problems in developing countries including Cameroon.

In Cameroon more specifically, similar studies have been carried out in many localities except in Bamendjou Subdivision where children tend to be preys of this intestinal helminthiasis. Many arguments plead in favor of the hypothesis that infections are more abundant in areas where we are confronted with problems such as a demographic explosion, multiplication of school without adequate provision of potable clean water, or management of feces disposal [5]. Given that Bamendjou is confronted with these difficulties, we can then assume that the population in general and school-age children in particular are potential targets of gastrointestinal helminths. The main objective was to provide information on the prevalence of these gastrointestinal parasites among school-age children in Bamendjou village in order to provide baseline information that could be exploited for the control of gastrointestinal parasites.

## 2. Material and Methods

### 2.1. Study Area

**Figure 1:**
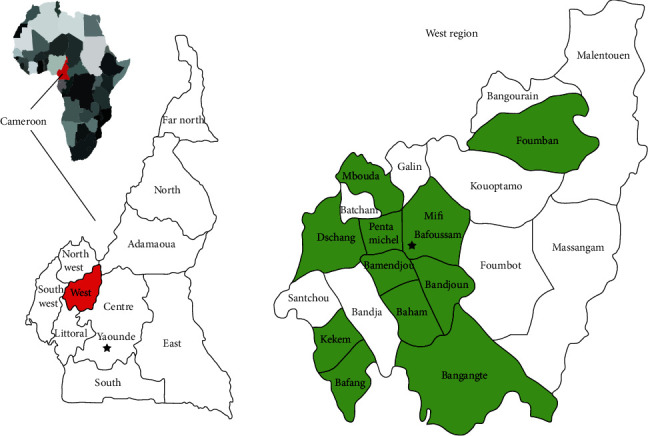
Map of the study area [6].

This study was carried out between March and September 2019 in the community of Bamendjou (Figure 1) which is found in the Upper Plateaux Division, in the West Region of Cameroon with a surface area of 250 km^2^. Bamendjou is situated at 5.39° North latitude, 10.33° East longitude, and 1537 meters elevation above sea level. Bamendjou is the Subdivisional Head Quarters with an overall population of 34269 inhabitants according to the 2005 Census and 5351 inhabitants for the town of Bamendjou.

### 2.2. Study Design

This was a descriptive study since it consisted of describing the health status of the population by measuring epidemiological factors such as prevalence and intensity of the different gastrointestinal helminths affecting school-age children. The informed consent forms alongside with questionnaires were distributed to the children to take home. Information on some risk factors for the acquisition of these gastrointestinal helminths (GIPs) was obtained using the prestructured questionnaire with questions relating to some social and behavioral habits. The children were educated on how to collect the stool samples in order to avoid contamination with urine and other objects. Stool samples (first stools of the morning) were collected the same morning by trained field workers alongside the principal investigator from the study participants in labelled plastic screw-cap vials with confidential information (codes such as sex, age, date, and number) and then transported to the University of Dschang for parasitological examination procedures in the Laboratory of the Research Unit of Biology and Applied Ecology (RUBAE) of the University of Dschang (UDs).

### 2.3. Study Population

The study population consisted of school-age children. Four hundred and ninety-three (493) children of ages between 3 and 22 years consisting of girls and boys from six schools were sampled: two nursery (Government Bilingual Nursery School Bamendjou (GBNS) and St. Theresa Nursery School Bamendjou Toba (STNS)), two primary (Ecole Publique du Centre Bamendjou (EPC) and St. Theresa Primary School (STPS)), and two secondary schools (Government Bilingual High School Bamendjou (GBHSB) and Collège Tankou Philippe Bamendjou (COTAPHI)).

### 2.4. Inclusion and Exclusion Criteria

The participants must understand the purpose of the study before providing stool samples and children of 2 years that participated; their parents or their guidance must understand the study before allowing them to participate. School-age children in the targeted schools willing to participate and have not been dewormed during a period of one month were included in the study. All those who were at least two years of age and above were included in the study. Those who signed the informed consent form and those whose parents signed for them were included in the study.

Those who did not give their consent, those that were recently enrolled in the schools, and those who were on anthelminthic treatment within a period of one month were excluded. Participants that did not provide stool samples but answer questionnaires were excluded.

### 2.5. Sample Collection

An oral description and specific instruction for handling and avoidance of contamination of the stool specimens (e.g., not to eat vegetables and charcoal) was given to all the subjects by the principal investigator. A structured questionnaire was used to collect data on the socio-demographic, clinical history of the participants and risk factors of intestinal helminth. The questionnaire was prepared in English and French and then translated into the local language for communication convenience. Questions were answered by respondents in the presence of parents for children. Prepared questionnaires were taken home for their parents to help them in answering the questions. Disposable labelled plastic cups or vials (with identification numbers which coded the name, age, time, and place of collection) with clean applicator sticks were distributed to all study subjects to collect fresh first stool specimens.

### 2.6. Microscopic Examination

Fecal samples were analysed using the simple salt floatation technique described by Thienpont et al. [7]. Briefly, a saturated solution of sodium chloride was prepared by measuring 400 g to 1000 ml of distilled water and mixed thoroughly in a clean 1500 ml measuring cylinder. This solution was allowed to stand for 48 hours (Cheesbrough, 1987). This solution was used in the floatation technique. The advantage of this salt floatation technique is that light infections are detected by this technique invariably, and only eggs are clearly visible without the hindrance of fecal and fiber material.

### 2.7. Morphological Identification and Quantitative Analysis

Identification of the parasitic eggs was aided by photos and dimension ranges prepared by the World Health Organization (WHO) Bench Aid for the Diagnosis of Intestinal Parasites and that of Thienpont et al. [7]. A quantitative analysis was carried out for the positive samples using the Mc Master Technique. With the aid of a Pasteur pipette, 0.1 ml of the suspension was drawn while stirring the mixture and then used to fill the cell one after the other in an inclined manner so as to avoid the creation of air bubbles. These counting chambers were allowed to stand for about 5 minutes to allow eggs to float to the surface and debris to sediment. The suspension was then examined under the microscope at 10x magnification. All eggs within the engraved region of both chambers were counted [7]. The number of eggs per gram (EPG) was calculated as follows:

(1) $$\begin{array}{c} N=\frac{\left(X_{1}+X_{2}\right)}{2}, \end{array}$$

where *X*_1_ is the number of eggs counted in the first chamber, *X*_2_ is the number of eggs counted on the second chamber, and *N* is the average number of eggs counted in both chambers.

Therefore, EPG = *N* × 200.

EPG = 50 when the eggs observed during floatation were no longer observed in McMaster.

### 2.8. Parameters Studied

#### 2.8.1. Prevalence

The prevalence (*P*) was calculated using the formula:

(2) $$\begin{array}{c} P=\frac{\text{Number of individuals infected}}{\text{Number of individuals examined}}\times 100. \end{array}$$

#### 2.8.2. Community Risk of Infection

Based on the prevalence of infection of the STHs, the community could be classified into low-risk, moderate-risk, and high-risk categories according to the WHO disease-specific thresh holds, and this classification can be used to determine the appropriate treatment regime as specified in the WHO guidelines (WHO, 2006) as follows: low-risk (<20%), moderate-risk (≥20 but <50%), and high-risk (≥50%).

#### 2.8.3. The Mean Parasitic Load or Intensity

The mean parasitic load was expressed in terms of mean egg per gram (EPG) of feces using the following formula: ∑EPG/*n*, where *n* is the number of infected individuals.

#### 2.8.4. Degree of Infection

The degree of infection was determined from the parasitic load (intensity). The World Health Organization (WHO) criteria were used to classify each infected sample as being of low, moderate, or high-intensity infection as seen in Table 1.

### 2.9. Statistical Analysis

Data obtained were entered and stored in a Microsoft Excel spread sheet and then exported to the SPSS (Statistical Package for Social Science, version 20.0) software for analyses. The prevalence of helminth infections was compared between sex, age groups, and schools using the chi-square test (*χ*^2^). This test was also used to determine the level of significance between parameters (infection and risk factors). They were all tested at 5% significance level (thresh holds of *P* < 0.05) and 95% confidence interval (CI).

## 3. Results

Out of the 493 stool samples examined, 57 (11.6%) were infected with at least one parasitic helminth (Table 2). Four helminths were recorded, all of which were nematodes. These nematodes included *Ascaris lumbricoides*, hookworm, *Trichuris trichiura*, and *Strongyloides stercoralis*.

It can be seen from Table 3 below that out of the 262 examined, 35 (13.1%) females were positive while out of the 231 males examined, 28 (12.2%) harbored parasites. It can be deduced that there is no significant difference between the prevalence of males and females (*P* > 0.05).

It can be seen from Table 4 that all age groups were infected with at least one parasite. Children aged 20 years and above had the highest prevalence of infection (*Ascaris lumbricoides* 30%, *Trichuris trichiura* 10%, and hookworm 10%) while the lowest prevalence was recorded in the age group above 6-10 years (8.7%). *Ascaris lumbricoides* showed a significant difference between the age groups (*P* < 0.05).

Table 5 presents the prevalence of parasitic helminths among schools. We observed from this table that *Ascaris lumbricoides* was the most prevalent in nursery, primary, and secondary schools. There was no significant difference (*P* > 0.05) between the level of education with all the parasites.

Table 6 presents the types of parasitic association. From this table, fifty-one (51) individuals had monoparasitism with a prevalence of 10.3%. Double parasitism (*A lumbricoides* + *T. trichiura*, *A. lumbricoides +* hookworm, and *T. trichuria +* hookworm) came from six (6) participants with 1.2% as prevalence.

**Figure 2:**
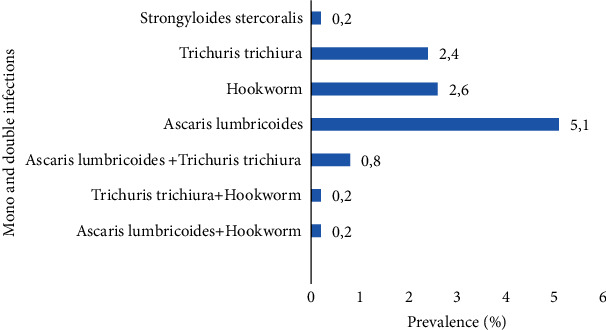
Prevalence of single and mixed parasitic infections.

Figure 2 below shows the patterns of the different types of parasitic association. *Ascaris lumbricoides* recorded the highest prevalence of 5.1% among the single infections. Concerning double infections (*Ascaris lumbricoides* + *Trichuris trichiura*) was the highest with 0.8%.

The specific intensity of infection which is expressed in terms of the mean concentration of eggs per gram (EPG) of feces is shown in Table 7. Out of the 493 stool samples examined, *Ascaris lumbricoides* had the highest intensity (2260 ± 6577.98) of infection while *Strongyloides stercoralis* (200 ± 00) had the lowest intensity of infection.

Table 8 shows the degree of infection. From this table, it appears that 61.90% of infection was light, 18.84% moderate, and 17.46% heavy. All three helminths (*Ascaris lumbricoides*, *Trchuris trichiura*, and hookworm) recorded light moderate intensities of infection with a prevalence of 40.6%, 80%, and 87.5%, respectively, for light intensity and 25%, 20%, and 12.5%, respectively, for the moderate intensity. Only *Ascaris lumbricoides* (34.4%) showed heavy intensity of infection.

Table 9 below outlines some of the risk factors together with the total infections. From this table, it is seen that only habits of not wearing shoes all the time and those who had stream as their source of drinking water showed significant relationships with the general nematode infections. Risk factors such as treating drinking water, not washing fruits always before eating or not at all, not washing hands after using the toilet, showed high degrees of infection though not significant.

## 4. Discussion

It can be seen from our study that all the schools were infected with at least one gastrointestinal helminth. These results were reported by Benzerroug [8] in Cape Verde and Ngangnang and Payne [1] in Cameroon. This could probably be due to a direct consequence of nonrespect of the basic individual hygiene and sanitation practices in schools as well as at home [9].

The overall prevalence of intestinal nematodes was 11.6% (57/493). This prevalence was lower than that of a previous study conducted by Tchouyabe [10], in Njimom in the Noun Division who had a 77.5% prevalence of helminth infections. It was also found to be lower (24.5%) than that observed by Ngangnang and Payne [1], in Nkondjock (Cameroon). Contrarily, this prevalence was higher than that observed by Sammy et al. [11] in the Ashanti Region of Ghana who had prevalence of 11.2%. These different levels of prevalence could be due to the behavioral habits and living standard of study participants or due to geographical condition of the study area [12].

In this study, it was observed that the prevalence of infection in nursery school children was 13.6%. This observation was low compared to 38.7% prevalence observed in Haiti by Charpetier et al. [13]. The prevalence was slightly higher in nursery school children than in the primary school children (10.2%). This could be due to the fact that most nursery school children are still ignorant of the importance of personal hygiene and sanitation. Although they are usually collectively taken care of in schools, individual habits still remain a major problem as most of them defecate on themselves and are commonly seen moving around barefooted and always in contact with the soil. In secondary school children, the prevalence was highest (20.1%). This confirms the observations of Ngangnang and Payne [1], in Cameroon, who all had cases of secondary school children dominating. This comparative high prevalence at this level of education could be due to the fact that these children being much older are usually left to take care of themselves, and most of them tend to neglect basic hygiene and sanitation principles. Negligence of basic hygiene is a great risk for contamination or infection since most often they are the ones to take care of the very young ones.

Four parasitic nematodes (*A. lumbricoides*, *T. trichiura*, hookworm, and *S. stercoralis*) were identified during this study with specific prevalence of 6.1%, 3.4%, 3.0%, and 0.2%, respectively. Similarly, these same helminths were observed by Glickman et al. [14] in Guinea among school children, but their prevalences were all higher. They observed a 35.7%, 18.9%, 12.2%, and 10.1% for hookworms, *A*. *lumbricoides*, *T. trichiura*, and *S. stercoralis*, respectively. Another study carried out by Al-Meckhalafi et al. [15] showed a prevalence of 8.5% for *A. lumbricoides* which agrees with that of this study (6.1%) and a much more lower rate of *T. trichiura* (0.5%) disagreeing with that of the present study.

The high prevalence of *Ascaris* infection could be attributed to the fact that the egg of *A. lumbricoides* can survive a prolonged period of 10 years under a warm, shady, and moist environmental condition which could be a reason for their long constant infection.

From the general population examined, it was seen that children aged 20 years and above had the highest prevalence (40.0%). Bismarck et al. [16] showed that infection was most pronounced among adult age groups. This could be due to the fact that older children lived a community life and were not observing rules of general and personal hygiene.

However, these results disagree with earlier findings by Menan et al. [17] and that of Ngangnang and Payne [1] who observed a higher prevalence instead in younger age groups. This could be explained by the fact that younger children are going through a period of rapid physical growth and rapid metabolism marked by an increase in their nutritional needs and by that do not control food intake [5].

Females were seen to show a higher prevalence (13.1%) for all parasitic worm species than the males (12.2%). The studies carried out by Ngangnang and Payne [1] did not agree with this observation since they had more males being infected than females. Meanwhile, studies carried out by Bareket and Zewdneh [18] in Ethiopia and Olawumi et al. [19] in Malaysia showed that females were more infected than males agreeing with the observation of this study. This higher prevalence shown by females in this area could be linked to the high levels of farming activities carried out in this area since they are mostly involved in farming than males, thus increasing their chances of being contaminated with the STHs.

The prevalence of double parasitism was 1.2% not far from that of the study of Ragunathan et al. [20] who observed a prevalence of 1.8% though does not agree with that of Menan et al. [17] who had a higher prevalence of 26.0%. The most abundant parasitic association was that of *Ascaris lumbricoides* + *T. trichiura* (0.8%) and could probably be due to the similarity between their routes of transmission (feco-oral) and also the occupation of sites that are not the same in the digestive tract [21].

*Ascaris lumbricoides* had the highest mean intensity (2260 ± 6577.98) among the nematodes recorded in the study. The mean intensity of *A. lumbricoides* recorded was slightly lower than that obtained by Wabo et al. [22] who registered 3722 ± 5677 for *A. lumbricoides*. This high intensity of *A. lumbricoides* could be due to the fact that *A. lumbricoides* has a higher egg output (200,000 eggs per day) compared to hookworm and *T. trichiura*.

The degree of infection due to *A. lumbricoides*, *T. trichiura*, and hookworm showed that the majority of infections were light (61.90%). This is in accordance with Mbu and Nembu [23], who recorded 100% light infection. *Ascaris lumbricoides* showed heavy infections while *T. trichiura* and hookworm showed moderate infection. Generally, helminth infections of moderate and high intensity in the gastrointestinal tract produce clinical manifestations.

The investigations revealed that only the risk factors, habits of not wearing shoes all the time and those who had stream as their source of drinking water, showed significant influence (*P* < 0.05) on the acquisition of intestinal parasitic worms, since not treating drinking water especially from wells and streams and the habit of wearing shoes at times showed high infections. In the same light, a study carried out by Blondo [24], in Banka, Cameroon, showed only one significant risk factor for worm infections. This was contrary to the observations of Sinar et al. [25] in Turkey who observed significance between all the risk factors and intestinal helminth infections. Several of these risk factors have been reported by authors in different countries [26, 27]. In such cases, promoting health education interventions targeting such behaviors especially personal hygiene and sanitation and provision of potable water are critically needed to augment the effectiveness of mass drug administration and to prevent infections from bouncing back after the termination of such campaigns [28].

## 5. Conclusion

The present study reveals that the health status of school going children is limited due to the relatively poor hygiene and sanitary conditions. This study has shown that intestinal nematodes are prevalent in varying intensities among school children in the community of Bamendjou. The relatively low overall prevalence obtained in this study shows that the national deworming campaigns are proving effective. In the future, we hope to carry out a retrospective study in order to determine the incidence of infections with parasitic worms, so as to evaluate the efficacy of preventive and control programmes. We equally hope to study the coinfection of gastrointestinal helminths and other parasites and their effects on the health status of the individuals.

## 6. Limitations of the Study

Due to resource constraints, we did not perform molecular techniques like PCR to identify the different intestinal helminth. And also, we did not perform other sensitive methods specific for some intestinal parasites such as Kato-Katz method, Trichome, and modifed Ziehl–Neelsen staining methods.

## Figures and Tables

**Table 1:** Thresholds for classification of STHs according to degrees of infection.

Parasites	Light (low) (EPG)	Moderate (EPG)	High (heavy) (EPG)
*A. lumbricoides*	1-4.999	5.000-9.999	≥10.000
*T. trichiura*	1-1.999	2.000-3.999	≥4.000
Hookworm	1-1.999	2.000-3.999	≥4.000

EPG: egg per gram of feces; STHs: soil-transmitted helminths.

**Table 2:** The overall percentage of infected and noninfected individuals.

Parasitic status	Number of individuals	Frequency (%)
Infected with at least one parasite	57	11.6
Noninfected	436	88.4
Total	493	100.0

**Table 3:** Prevalence of parasitic helminths according to gender.

	Gender					
Parasitic helminths	Females	Males	Total	*Χ* ^2^	Df	*P* value
	*N* (%) Inf	*N* (%) Inf	*N* (%)Inf			
*Ascaris lumbricoides*	16 (6.1)	14 (6.1)	30 (6.1)	0	1	0.99
*Trichuris trichiura*	9 (3.4)	8 (3.5)	17 (3.4)	0	1	0.98
Hookworm	9 (3.4)	6 (2.6)	15 (3.0)	0.29	1	0.58
*S. stercoralis*	1 (0.2)	0	1 (0.2)			
Total	35 (13.1)	28 (12.2)	63 (12.7)			

**Table 4:** The prevalence of helminth infection according to age group.

	Parasitic helminths			
Age group (years)	*Ascaris lumbricoides*	*Trichuris trichiura*	Hookworm	Total
	*N* (%)	*N* (%)	*N* (%)	*N* (%)
<05	8 (11.4)	2 (2.9)	4 (5.7)	14 (20.00)
06-10	7 (4.7)	4 (2.7)	3 (2)	13 (8.7)
11-20	12 (4.6)	10 (3.8)	7 (2.7)	26 (9.9)
>20	3 (30)	1 (10)	1 (10)	4 (40)
Total	30 (6.1)	17 (3.4)	15 (3)	57 (11.6)
*χ* ^2^	15.1	1.73	4.02	14.74
*P* value	0.02	0.63	0.26	0.002

**Table 5:** The prevalence of parasitic helminths among schools.

	Different schools					
	Nursery	Primary	Secondary	Total	*X* ^2^	*P* value
Parasites	*N* = 68	*N* = 199	*N* = 226	*N* = 493		
*Ascaris lumbricoides*	8 (11.7%)	9 (4.6%)	13 (5.9%)	30 (6.1%)	5.1	0.4
*Trichuris trichuria*	3 (5%)	4 (2.5%)	10 (4.4%)	17 (3.4%)	10.56	0.06
Hookworm	2 (3.4%)	4 (1.9%)	7 (3.3%)	15 (3%)	3.72	0.59
*Strongyloides stercoralis*	—	1 (1.2%)	—	1 (0.2%)	—	—
Total	13 (13.6%)	18 (10.2%)	30 (20.1%)			

**Table 6:** Type of parasitism.

	Females	Males	Total
	*N* = 262	*N* = 231	*N* = 493
Total number of children infected	32 (56.1%)	25 (43.9%)	57 (11.6%)
Monoparasitism	29 (56.9%)	22 (43.1%)	51 (10.3%)
Double parasitism	3 (9.4%)	3 (12.0%)	6 (1.2%)

**Table 7:** Intensity of infection of helminth parasites.

Parasites	No. infected	Egg per gram (mean ± SD)
*Ascaris lumbricoides*	30	2260 ± 6577.98
*Trichuris trichiura*	17	223.53 ± 264.054
Hookworm	15	416.67 ± 427.061
*Strongyloides stercoralis*	1	200 ± 0

**Table 8:** Degree of infection.

	Intensity of infection			
Parasites	Light *N* (%)	Moderate *N* (%)	Heavy *N* (%)	Total *N*
*Ascaris lumbricoides*	13 (40.6)	8 (25.0)	11 (34.4)	32 (100)
*Trichuris trichiura*	12 (80.0)	3 (20.0)	—	15 (100)
Hookworm	14 (87.5)	2 (12.5)	—	16 (100)
Total	39 (61.90)	13 (18.84)	11 (17.46)	63 (100)

**Table 9:** Association between potential risk factors and helminth infection among school children in Bamendjou.

	*N*	Infected	Noninfected	OR (95% CI)	*P* value
Wearing shoes					
Always	476	52	424	1	NA
At times	16	5	11	3.71 (1.23-11.08)	0.04
Washing feet					
Always	298	37	261	1	NA
At times	191	20	171	0.82 (0.46-1.47)	0.51
Never	4	0	4	0.77 (0.04-14.68)	0.86
Hand washing					
Never	14	2	12	1.24 (0.26-5.99)	0.78
No soap	226	21	205	0.76 (0.4-1.47)	0.42
Soap	161	19	142	1	NA
					
Water source					
Tap	287	25	262	1	NA
Stream	121	19	102	1.95 (1.03-3.7)	0.04
Tap/stream	16	2	14	1.49 (0.32-6.96)	0.61
Well	18	1	17	0.61 (0.08-4.93)	0.64
Treating water before drinking					
Yes	9	1	8	1	NA
No	36	4	32	1 (0.09-10.22)	1

NA: not applicable.

## Data Availability

Data and material are available to other researchers upon request.

## Abbreviations

EPG: Egg per gram of feces
GBNS: Government Bilingual Nursery School Bamendjou
STNS: Saint. Theresa Nursery School Bamendjou Toba
EPC: Ecole Publique du Centre Bamendjou
STPS: Saint Theresa Primary School
GBHSB: Government Bilingual High School Bamendjou
COTAPHI: Collège Tankou Philippe Bamendjou.
